# Physico-Chemical and Mineral Variability of *Apis mellifera* Bee Venom Across Seasons and Feeding Regimes

**DOI:** 10.3390/molecules31111834

**Published:** 2026-05-26

**Authors:** Adrian-Dan Rășinar, Isidora Radulov, Adina Berbecea, Silvia Pătruică

**Affiliations:** 1 Faculty of Bioengineering of Animal Resources, University of Life Sciences “King Mihai I” from Timisoara, Calea Aradului No. 119, 300645 Timisoara, Romania; adrian.rasinar@usvt.ro (A.-D.R.); silviapatruica@usvt.ro (S.P.); 2 Faculty of Agriculture, University of Life Sciences “King Mihai I” from Timisoara, Calea Aradului No. 119, 300645 Timisoara, Romania

**Keywords:** *Apis mellifera*, bee venom, supplemental feeding, climate change

## Abstract

Bee venom variability is driven by environmental and nutritional factors, yet their integrated effects remain poorly understood. This study provides a novel, comprehensive assessment combining dietary treatments with real-time environmental monitoring to evaluate their joint influence on the physico-chemical properties, total amino acid, mineral composition, and heavy metal content of *Apis mellifera* venom. A total of 32 samples collected between April and July 2025 were analyzed under both artificial feeding and natural foraging conditions. Moisture ranged from 11.2% to 19.2%, while pH remained stable (5.6–6.25). Total amino acids varied between 344.0 and 409.5 mg/g, with maximum values during the acacia period (>400 mg/g). Potassium was the dominant macroelement (3.19–11.37 mg/g), followed by Ca (0.80–3.68 mg/g) and P (0.31–1.84 mg/g). Microelements such as Fe (0.11–0.98 mg/g) and Mn (1.19–8.85 µg/g) showed pronounced seasonal variability. Lead reached up to 36.18 µg/g during natural foraging, while Cd (0.30–3.97 µg/g) was mainly associated with artificial feeding. By integrating nutritional and microclimatic determinants, this study demonstrates that floral origin and seasonal dynamics are the primary drivers of venom quality, while supplementation exerts secondary effects, and highlights the potential of bee venom as a sensitive bioindicator of environmental exposure.

## 1. Introduction

Bee products occupy a central position in contemporary research on natural bioactive compounds and in alternative medicine. Among these, bee venom (apitoxin) is regarded as one of the most promising biological products due to its complex chemical structure and diverse pharmacological activities [1,2,3]. Traditionally used in apitherapy, bee venom is recognized for its anti-inflammatory, analgesic, antimicrobial, and immunomodulatory properties and has been investigated as a therapeutic agent for rheumatoid arthritis, multiple sclerosis, neurological disorders, and certain cancers [4,5,6].

Bee venom is secreted by worker bees and contains more than 200 bioactive molecules, including peptides (melittin, apamin, adolapin), enzymes (phospholipase A_2_, hyaluronidase), biogenic amines, sugars, and a broad spectrum of macro- and microelements [7,8,9]. Its composition is shaped by multiple intrinsic and extrinsic factors, such as bee age and race [10], colony health, season [11], climatic conditions, available flora [12], and especially the nutritional regime [13,14]. While the major protein and enzymatic components of venom are well documented, information on its mineral composition and antioxidant capacity remains fragmented and difficult to compare across studies [15,16,17].

In recent decades, beekeeping has been challenged by biodiversity loss, intensive pesticide use, climate change, and habitat degradation. These pressures have affected colony health and productivity, prompting widespread use of supplemental feeding strategies—including sugar syrup, essential oils [18], and probiotic formulations—to compensate for resource shortages, support immune function, and enhance the quality of bee products [12,19]. Although numerous studies have demonstrated the positive effects of dietary supplements on honey, wax, royal jelly, and propolis production, their influence on bee venom quality remains insufficiently explored [13]. Emerging evidence suggests that supplements based on essential oils (e.g., oregano, thyme, basil) may affect not only venom yield but also its biochemical composition and antioxidant properties [20,21,22,23]. Given the antimicrobial, antifungal, and antioxidant activities of these oils, their incorporation into colony diets may transfer functional benefits to secreted products, including venom.

Probiotic supplements, such as lactobacilli and bifidobacteria, are increasingly used to stabilize the gut microbiota, reduce oxidative stress, and prevent opportunistic infections [24,25]. Improved colony health may, in turn, influence venom biosynthesis, although experimental confirmation of this relationship remains limited. Additionally, the botanical origin of forage (e.g., rapeseed, acacia, sunflower) may alter the natural antioxidant compounds and mineral elements incorporated into venom.

A further dimension of venom quality relates to the accumulation of heavy metals, including Pb, Cd, Cu and Zn. As pollinators, bees interact directly with air, water, and soil, making their biological products effective indicators of environmental contamination [17]. The presence of toxic metals in venom may therefore reflect local pollution levels and has implications for both ecotoxicological assessment and the safety of venom-based therapies [26].

Bee venom composition is increasingly recognized as dynamic rather than static. Seasonal variation, forage availability, and dietary inputs significantly influence its chemical profile [3,4,5], reflecting adaptive colony responses to fluctuating environmental conditions. However, this variability complicates efforts to standardize venom for research or therapeutic applications. Seasonal cycles affect both colony physiology and the abiotic environment, with temperature fluctuations modulating metabolic activity and altering the synthesis and relative abundance of venom components [6,7,8,9,10]. Simultaneously, the phenology and diversity of flowering plants determine the nutritional resources available to foragers, directly influencing the proteins, amino acids, and precursor molecules required for venom biosynthesis [11,12,13,14,15]. Beekeeping practices such as supplemental feeding further modify these nutritional inputs and may affect worker physiology in ways that influence venom production [19,20,21,22]. Despite these insights, the combined effects of seasonality, forage quality, and nutrition—particularly on the enzymatic, mineral, and elemental profiles of venom—remain insufficiently understood.

In Romania, studies examining meteorological influences on apicultural productivity have been sporadic, and research directly linking venom yield or quality to temperature and humidity during harvesting is notably scarce. This gap is especially relevant in the context of climate change, which increasingly alters local microclimatic conditions.

The present study investigates how different nutritional regimes (sugar syrup, essential oils, probiotic supplements, and natural pollen–nectar sources) shape the biochemical composition, mineral profile, and heavy metal accumulation of *Apis mellifera* venom. In parallel, we evaluated the influence of temperature and humidity on venom composition, considering their role in regulating foraging activity, colony metabolism, and the biochemical processes underlying venom biosynthesis. By integrating nutritional and microclimatic determinants, this work aims to clarify how environmental and management factors interact to determine venom quality and to support the development of optimized beekeeping practices.

## 2. Results and Discussion

The analysis of the physico-chemical parameters (mean values ± SD) (Table 1) of bee venom highlighted significant variations depending on the harvesting period, food source, and type of supplement administered to the colonies.

### 2.1. Dry Matter and Moisture

The dry matter content of the samples varied from 80.8% (V2-Rnp2) to 88.8% (V6-Rnp6), corresponding to moisture levels of 11.2–19.2%, an interval that reflects the expected inverse relationship between these two parameters. These values align with those typically reported for dehydrated bee venom, indicating an advanced dehydration state that supports the stability and long-term preservation of its bioactive constituents [27,28].

Comparative analysis by experimental variants and harvesting periods shows that the minimum moisture was recorded in sample V6-Rnp6 (11.2 ± 0.3%), corresponding to rapeseed flow, while the maximum value was observed in V2-Rnp2 (19.2 ± 0.5%). Samples from April (Ss), obtained under artificial feeding conditions with sugar syrup and supplements (essential oils or probiotics), showed reduced variability and moderate moisture values, while samples from natural foraging periods (rapeseed, acacia, sunflower) showed more pronounced variability. This variability is consistent with studies showing that venom properties are influenced by biological and environmental factors, including food source and season [29,30].

The consistent inverse association between moisture and dry matter confirms the characteristic behavior of bee venom during dehydration. The observed patterns suggest that seasonal dynamics and the botanical origin of nectar exert a stronger influence on these parameters than nutritional supplementation, a trend also documented in studies addressing venom compositional variability [27,31].

Low moisture combined with elevated dry matter points to enhanced product stability and a lower susceptibility to enzymatic degradation, conditions essential for maintaining the biological integrity of key components such as melittin and other active peptides [32,33]. The greater variability recorded during natural foraging periods is plausibly linked to climatic effects on colony metabolism and venom secretion, as previously reported [29].

In contrast, high moisture values, as in the case of V2-Rnp2, may indicate greater water retention, determined by environmental conditions or nectar characteristics. Overall, the results confirm that bee venom moisture is predominantly influenced by ecological and physiological factors, while nutritional interventions have a limited effect on these parameters [28,30].

### 2.2. Venom pH

The pH values of bee venom samples showed low variability, ranging between 5.6 and 6.25, confirming the slightly acidic nature of this biological product. The lowest value was recorded in variant V6-Rnp6 (5.6 ± 0.06), corresponding to rapeseed flow, while the maximum value was observed in variant V1-Ss (6.25 ± 0.05), associated with the artificial feeding period. These results are consistent with literature describing bee venom as having a slightly acidic pH, associated with the stability and biological activity of its components [7,31].

The distribution of values by harvesting period shows a clear trend: samples obtained under syrup feeding conditions (Ss) showed slightly higher pH values (approx. 6.0–6.25). Although pH values varied across rapeseed samples, the minimum pH observed in the entire dataset occurred during the rapeseed foraging period (V6-Rnp6, pH 5.6). Samples from acacia (Anp) and sunflower (Snp) generally fell within intermediate ranges, maintaining the same slightly acidic character. This variation may reflect differences in the chemical composition of the venom, indirectly influenced by food sources and seasonal conditions [29,30].

Despite these fluctuations, pH remained relatively constant across experimental variants, including under conditions of supplementation (essential oils and probiotics) during artificial feeding. The lack of systematic changes suggests that venom acidity is a stable parameter, predominantly controlled by internal physiological mechanisms of bees rather than external nutritional factors. This aspect is supported by studies indicating that the fundamental physico-chemical properties of venom are strictly biologically regulated [27,33].

From a biochemical perspective, the pH of bee venom is known to reflect the contribution of acidic peptides (such as melittin and apamin) and free amino acids, which collectively shape its overall acidity [32,34]. The subtle variations reported between samples in the literature are generally attributed to minor compositional shifts and do not alter the fundamental characteristics of the venom.

In this context, the relative stability of pH across treatments and harvesting periods—consistently documented in previous studies—indicates that this parameter is largely governed by species-specific physiology rather than by dietary factors or supplementation, remaining within the characteristic limits described for bee venom [28,31].

### 2.3. Ash Content

The ash content of bee venom samples ranged between 3.1% and 5.5%, reflecting differences in their mineral composition. The maximum value was recorded in variant V4-SsO (5.5 ± 0.4%), corresponding to supplementation with oregano essential oil, while the minimum values were observed in V2-Rnp2 (3.1 ± 0.3%) and V8-Anp8 (3.2 ± 0.3%). These variations indicate moderate heterogeneity of the mineral fraction, consistent with literature data on bee venom composition [28,31].

Analysis by experimental variants shows that samples obtained under artificial feeding conditions with syrup and supplemented with essential oils (SsB, SsT, SsO) generally had slightly higher ash content values compared to samples from natural foraging. In particular, the SsO variant suggests a possible influence of supplementation on mineral metabolism or venom composition, although this trend is not uniform across all treatments. In contrast, samples from natural foraging (rapeseed, acacia, sunflower) showed greater variability but remained within the range of 3.1–5.3%, without a clear pattern exclusively associated with nectar type.

Ash content represents the inorganic residue of bee venom and is primarily determined by the mineral profile available to the colony through its trophic resources and local environmental conditions. Previous studies indicate that these external factors contribute to natural fluctuations in the mineral fraction of venom, explaining the modest differences observed between harvesting periods and experimental settings [29,35].

In our dataset, however, the ash values remained within the characteristic range reported for bee venom and showed no consistent or substantial response to supplementation. This pattern highlights the overall stability of the inorganic fraction, suggesting that mineral incorporation into venom is largely governed by species-specific physiological mechanisms, with nutritional and ecological influences introducing only limited, secondary variation [27,28].

### 2.4. Total Amino Acid Content

The total amino acid content of bee venom samples showed significant variations, ranging between 344 mg/g (V3-SsT) and 409.5 mg/g (V1-Anp1), highlighting an important influence of harvesting conditions on this parameter. The maximum values were consistently recorded in samples from the acacia foraging period (Anp), where most determinations exceeded the threshold of 400 mg/g, including V8-Anp8 (408.1 ± 0.9 mg/g).

This trend suggests that natural acacia nectar and pollen resources provide optimal nutritional input, favoring the synthesis of proteins. In contrast, lower values observed in some variants from the artificial feeding period, especially in the case of supplementation with essential oils (V3-SsT—344 mg/g), may indicate possible metabolic or stress effects on colonies, reflected in decreased protein biosynthesis capacity.

Comparative analysis by botanical source shows a clear pattern: samples from acacia (June) recorded the highest amino acid concentrations, followed by sunflower (July), while rapeseed (May) samples generally showed lower values. This gradient may be associated with differences in the nutritional quality of nectar and pollen, as well as seasonal physiological characteristics of bees [29,30].

Amino acid content is a key indicator of venom quality, as its main active components—melittin, apamin, and phospholipase A_2_—are protein-based and drive its pharmacological effects [32,36]. Higher levels therefore indicate greater synthesis of these compounds and superior biological quality. The obtained results indicate that supplementation with essential oils or probiotics does not lead to a systematic increase in amino acid content, while botanical origin and seasonal conditions represent the major determining factors of this parameter. These observations are consistent with the literature, which emphasizes the role of natural nutrition and colony physiological state in defining the biochemical composition of bee venom [27,37].

### 2.5. Macroelement Content in Bee Venom

The analysis of macroelement content (mean values ± SD) (Ca, K, Mg, and P) in bee venom (Table 2), based on the determined values (mg/g), highlights moderate yet biologically relevant variations depending on season and the type of supplements administered to the colonies. The obtained mineral profile indicates the predominance of potassium (K) in most analyzed variants, followed by calcium (Ca), phosphorus (P), and magnesium (Mg), a trend consistent with data reported in the literature [38].

Calcium concentrations showed moderate differences among samples, ranging from 0.806 ± 0.143 mg/g (V7-Snp7) to 3.686 ± 0.276 mg/g (V2-Anp2). A clear seasonal pattern is evident, with maximum values recorded during the acacia flowering period (June). For example, in variant V1, Ca content increased from 1.809 ± 0.341 mg/g (April, Ss) to 2.526 ± 0.17 mg/g (June, Anp1). In contrast, samples collected in July, (sunflower) frequently showed lower values (1.486 ± 0.248 mg/g in V1 and 1.626 ± 0.345 mg/g in V5). The influence of supplements was relatively limited; essential oils maintained slightly higher Ca levels (V4: 1.707 ± 0.376 mg/g) compared to probiotics (V5: 0.830 ± 0.021 mg/g). These results suggest that Ca variation is primarily driven by floral source, as also reported by Bogdanov (2016) [38].

Potassium exhibited the widest range of values, from 3.195 ± 0.251 mg/g (V7-Rnp7) to 11.372 ± 0.405 mg/g (V8-Anp8). A consistent increase in concentration was observed during the acacia period, reflecting the superior mineral input of this nectar source. Thus, in variant V3, K increased from 6.839 ± 0.209 mg/g (April) to 11.059 ± 0.497 mg/g (June). In contrast, July samples showed lower values (e.g., 3.320 ± 0.431 mg/g in V2-Snp2). Supplements had a secondary effect: essential oils resulted in moderate values (V3: 6.839 ± 0.209 mg/g), while probiotics were associated with lower concentrations (V7: 4.820 ± 0.198 mg/g). The predominance of potassium and its seasonal variability are consistent with other studies on venom composition [32].

Magnesium occurred in relatively low concentrations but displayed pronounced variation across samples, ranging from 0.100 ± 0.038 mg/g in V8–Anp8 to 0.951 ± 0.161 mg/g in V1–Anp1. As observed by several other elements, the highest Mg levels were recorded in June, a period typically associated with richer and more diverse floral resources. In variant V1, concentrations increased from 0.171 ± 0.011 mg/g (Ss) to 0.951 ± 0.161 mg/g (Anp1), a pattern that most plausibly reflects seasonal shifts in nectar and pollen mineral composition and environmental conditions influencing mineral uptake. July samples showed lower values, with the minimum of 0.127 ± 0.017 mg/g in V3–Snp3, consistent with the natural decline in resource diversity later in the season. Unlike Ca and K, Mg appeared more responsive to the type of supplement: essential oils were associated with moderately elevated levels (V4: 0.265 ± 0.075 mg/g), while the probiotic + oregano combination (V8) produced a more pronounced increase (0.671 ± 0.051 mg/g). These differences likely reflect supplement–driven changes in mineral assimilation or foraging behavior, reinforcing the dominant role of environmental and dietary inputs in shaping Mg content.

Phosphorus showed a narrower range of values (0.314–1.848 mg/g), with the highest concentrations recorded during the acacia period (1.848 ± 0.154 mg/g in V2-Anp2). Its relative stability compared to Ca and K indicates tighter metabolic control, given its involvement in essential energy processes. These trends are similar to those reported by Banks & Shipolini (1986) [39].

The macroelement content of bee venom, determined following the administration of the four types of feed, showed the predominance of potassium, followed by calcium, magnesium, and phosphorus. Similar results, but with lower values, were reported by Sabo et al. (2024) [26] for venom samples from different regions of Slovakia, where concentrations ranged between 0.366 and 1.140 mg/g for Ca, 1.221 and 4.005 mg/g for K, and 0.201 and 0.948 mg/g for Mg. The differences observed compared to the present study may be explained by the geographical and pedological characteristics of the study area, which influence floral composition and, consequently, mineral intake. Additionally, bee diet—including nectar and pollen sources, as well as administered supplements—along with environmental factors (temperature, humidity, general climatic conditions) may significantly contribute to variations in the mineral composition of venom.

The results highlight a clear seasonal pattern: the highest macroelement concentrations were recorded during the acacia flowering period (June), while the lowest values were associated with the sunflower period (July). Samples from the rapeseed period (May) showed intermediate but sometimes elevated levels, particularly for Ca and K. These trends confirm the determining role of floral resources in venom composition, consistent with observations reported by Bogdanov (2016) [38].

Regarding the influence of supplements, their effect on the mineral profile was differentiated but of moderate intensity. Essential oils led to slight increases in Ca, K, and Mg concentrations, while probiotics selectively influenced mineral absorption, resulting in differences among experimental variants. The probiotic + oregano combination produced element-dependent effects, more evident for potassium and magnesium. These findings are consistent with the literature highlighting the role of gut microbiota in nutrient assimilation and metabolism in bees [40].

The K/Ca ratio >1 in most samples confirms the predominance of potassium and its essential role in maintaining osmotic balance and cellular function [6,41]. Calcium contributes to the structural stability of protein and enzymatic components of venom [42], while magnesium acts as an essential enzymatic cofactor in numerous metabolic processes [43]. Phosphorus, relatively constant, reflects the energetic metabolism of colonies, being directly involved in the structure and function of ATP molecules [44]. The importance of these macroelements in bee venom is confirmed by recent studies highlighting their role in the biological activity of apitoxin. Compared to the literature [32,39], the obtained values fall within the reported ranges, confirming the validity of the results and the determining influence of nutritional and seasonal factors on the composition of bee venom.

### 2.6. Microelement Content in Bee Venom

**Figure 1:**
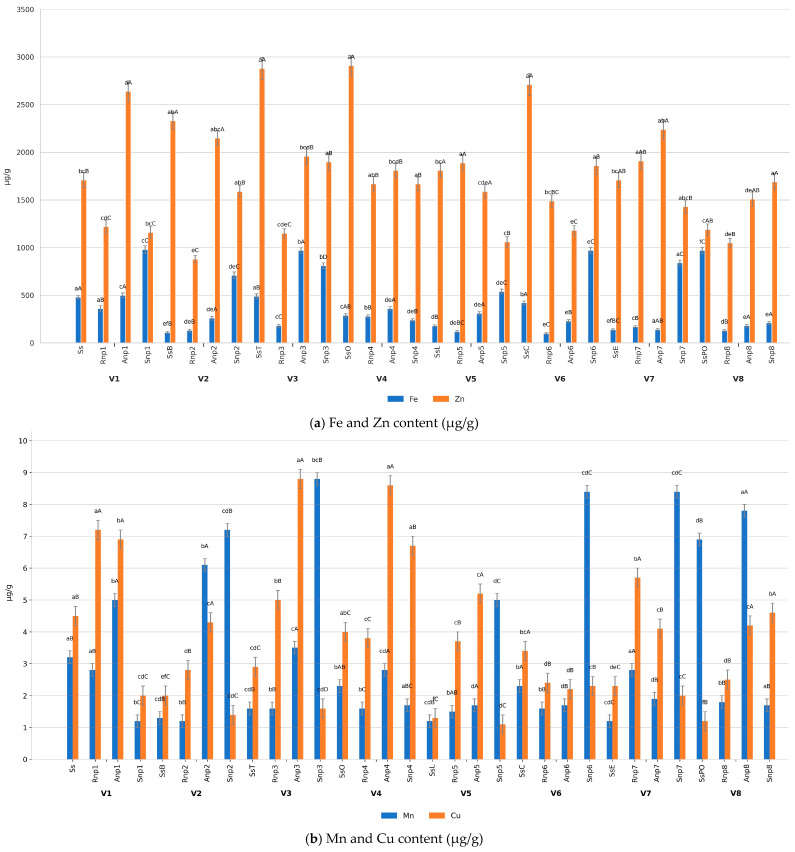
Microelement content (**a**—Fe and Zn content; **b**—Mn and Cu content). Means marked with the same letters show no statistically significant differences (*p* > 0.05), while means associated with different letters demonstrate statistically significant differences (*p* < 0.05). Lowercase letters are used to denote statistically significant differences between groups within the same harvest, whereas uppercase letters indicate statistically significant differences between harvests.

The analysis of microelement (mean values ± SD) (Figure 1) content (Fe, Mn, Cu, and Zn) in bee venom reveals significant variations depending on both the type of administered feed and the natural foraging source (rapeseed, acacia, sunflower). Figure 1 presents clear and structured patterns: (1) essential oils and probiotics differently modulate mineral absorption, and (2) natural foraging sources introduce strong seasonal and botanical variations.

Iron (Fe) content showed substantial differences, influenced by both supplements and floral origin. Under artificial feeding conditions (April), the control variant (V1-Ss) recorded 0.480 ± 0.023 mg/g, while essential oil treatments produced divergent responses: thyme (V3: 0.500 ± 0.015 mg/g) maintained similar values, whereas basil (V2: 0.117 ± 0.016 mg/g) and oregano (V4: 0.292 ± 0.027 mg/g) significantly reduced Fe concentration. Among probiotics, Colobiotic (V6: 0.429 ± 0.047 mg/g) and the probiotic–oregano combination (V8: 0.978 ± 0.022 mg/g) increased Fe levels, while Lacium (V5: 0.186 ± 0.074 mg/g) and Enterolactis (V7: 0.148 ± 0.077 mg/g) resulted in lower values. Under natural conditions, the highest Fe levels were recorded in sunflower (Snp1: 0.981 ± 0.088 mg/g; Snp6: 0.976 ± 0.080 mg/g) and acacia (Anp3: 0.980 ± 0.018 mg/g), while rapeseed showed lower values (e.g., Rnp1: 0.382 ± 0.024 mg/g).

Manganese (Mn) showed a strong dependence on the floral source. In April, values were generally low (e.g., V1: 3.267 ± 0.190 µg/g; V3: 1.604 ± 0.128 µg/g), with the exception of V8 (6.900 ± 0.420 µg/g). Under rapeseed feeding, Mn consistently remained low (≈1.191–2.803 µg/g), whereas acacia led to significant increases (Anp2: 6.066 ± 0.258 µg/g; Anp8: 7.854 ± 0.717 µg/g). The highest values were recorded in sunflower samples (Snp3: 8.849 ± 0.206 µg/g; Snp6: 8.338 ± 0.581 µg/g; Snp7: 8.343 ± 0.148 µg/g), highlighting the major influence of botanical origin on this element.

For copper (Cu), the results indicate a combined effect of supplements and nectar source. Under artificial feeding, essential oils increased Cu concentrations (V3: 2.917 ± 0.356 µg/g; V4: 4.022 ± 0.407 µg/g), while probiotics generally resulted in lower values (V5: 1.303 ± 0.442 µg/g; V8: 1.225 ± 0.316 µg/g). Under natural conditions, the highest values were observed in acacia (Anp3: 8.819 ± 0.372 µg/g; Anp4: 8.610 ± 0.615 µg/g), followed by rapeseed (Rnp1: 7.256 ± 0.409 µg/g; Rnp7: 5.675 ± 0.426 µg/g), whereas sunflower samples generally showed lower values (Snp1: 1.951 ± 0.143 µg/g; Snp2: 1.451 ± 0.122 µg/g).

Zinc (Zn) concentrations showed moderate differences, being influenced by both analyzed factors. In April, essential oils increased Zn levels (V3: 2.883 ± 0.255 mg/g; V4: 2.905 ± 0.116 mg/g) compared to the control (V1: 1.710 ± 0.111 mg/g), while probiotics had variable effects (V6: 2.706 ± 0.256 mg/g; V8: 1.194 ± 0.149 mg/g). Rapeseed feeding resulted in lower values (Rnp2: 0.879 ± 0.038 mg/g), while acacia showed higher values (Anp1: 2.640 ± 0.130 mg/g), and sunflower exhibited intermediate levels (Snp3: 1.897 ± 0.314 mg/g).

During spring, feed supplementation led to noticeable changes in the mineral profile. Variant V8 (probiotic + oregano) showed high Mn (6.900 ± 0.420 µg/g) and Fe (0.978 ± 0.022 mg/g) levels, suggesting a synergistic effect on mineral absorption. In contrast, the basil variant (V2) exhibited the lowest values for Fe (0.117 ± 0.016 mg/g) and Cu (1.974 ± 0.150 µg/g), indicating a possible inhibitory effect of volatile compounds.

Under natural foraging conditions, acacia favored the accumulation of Cu and Zn (e.g., Cu = 8.819 ± 0.372 µg/g in Anp3), while sunflower promoted high Mn accumulation (up to 8.849 ± 0.206 µg/g in Snp3) but lower Cu levels. These results are consistent with findings by Rășinar et al. (2025) [45], who reported Zn predominance followed by Fe, Cu, and Mn, as well as with studies by Sabo et al. (2024) [26] and Choińska et al. (2021) [17], which indicate similar ranges for these microelements.

Compared to the literature, the obtained values fall within reported ranges, although the differences are more pronounced, highlighting the role of dietary supplements. Studies by Bogdanov (2016) [38] and Hider (1988) [32] emphasized the influence of diet and environment, and our results complement these findings by demonstrating the specific effects of essential oils and probiotics. Additionally, Khalil et al. (2021) [46] showed that probiotics can enhance nutrient absorption, which is partially confirmed by the elevated values observed in combined treatments.

Our results (Table S1 in the Supplementary Materials) demonstrate that both supplemented artificial feeding and foraging type significantly influence the microelement content of bee venom. These variations may have important implications for the therapeutic properties of venom, and further studies are needed to elucidate the underlying biochemical mechanisms and optimize nutritional strategies in beekeeping. The data indicate that natural nectar sources exert a stronger influence on venom microelement composition than supplemental feeding, with acacia and sunflower nectar producing the most pronounced increases in Mn, Fe, and Cu. Essential oils modulate mineral absorption in distinct ways—thyme and oregano tend to increase Fe, Cu, and Zn levels—while probiotics generally reduce Cu and Zn but may increase Mn when combined with oregano. These patterns highlight the complex interaction between diet composition, botanical origin, and mineral metabolism in bee venom production.

The obtained results indicate that venom secretion is a physiologically robust and relatively conserved process, showing considerably lower sensitivity to short-term dietary interventions than other bee products. Venom gland activity is largely governed by intrinsic factors—such as worker age, seasonal stage, and foraging conditions [31,38] which can attenuate or even override subtle nutritional influences, particularly in healthy colonies with abundant floral resources.

The pronounced seasonal patterns observed in this study—reflected in the marked increases in amino acids and mineral elements during the acacia flow and in the distinct profiles recorded during rapeseed and sunflower periods—likely masked any minor effects of supplementation. These findings reinforce the view that natural floral resources are the primary determinants shaping venom composition [26,28,29,36]. Moreover, the influence of supplements administered during the artificial feeding period was probably reduced once colonies transitioned to natural foraging, consistent with literature showing that nectar and pollen play a decisive role in defining the biochemical characteristics of bee products.

The limited response to supplementation observed here may also be attributed to the experimental context, which involved healthy colonies, moderate supplement doses, and high availability of natural forage. In contrast, more pronounced effects reported in previous studies often arise under conditions of colony stress, higher supplement concentrations, or longer administration periods, which may explain the discrepancies with some earlier findings.

### 2.7. Heavy Metal Content in Bee Venom

**Figure 2:**
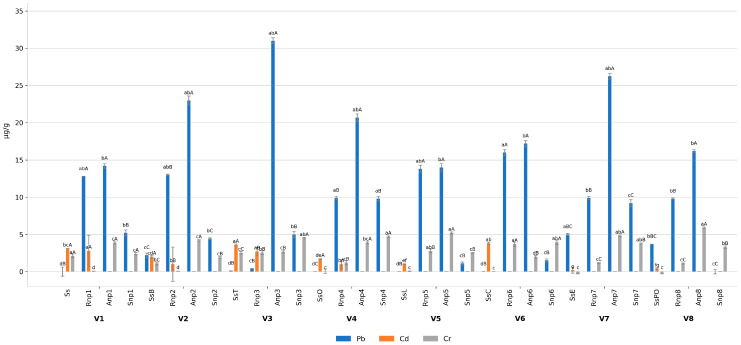
Pb, Cd, and Cr content in bee venom.

The analysis of Pb, Cd, and Cr (Table S2 in the Supplementary Materials) content in bee venom (mean values ± SD) (Figure 2) revealed significant variations determined both by the harvesting period and the type of feeding. Lead (Pb) was the predominant metal, with a wide concentration range (0–36.176 µg/g), indicating a major influence of the foraging environment. The lowest values (nd) were recorded in April in the Ss, SsT, SsO, SsL, and SsC variants, confirming the absence of Pb in sugar syrup and the administered supplements. In contrast, maximum values exceeding 30 µg/g were associated with natural foraging, especially during the acacia period (Anp3: 36.176 ± 5.662 µg/g). Considering that the maximum allowable dose of Pb in products used for medicinal purposes is 5 µg/day, and the usual dose of venom is 100 µg/day, the lead content determined in the venom samples does not pose a risk for its use [47]. In the control variant (V1), Pb increased from nd (Ss) to 11.990–13.333 µg/g during the rapeseed and acacia periods, highlighting the dominant role of environmental exposure. In supplemented April samples, Pb was detected only in SsB (2.471 µg/g), SsE (4.901 µg/g), and SsPO (3.373 µg/g), values much lower than those from natural foraging. In May, Pb was present in all samples (9–16 µg/g), while in June it reached the highest levels, followed by a decrease in July (1–10 µg/g; Snp5: 1.155 ± 0.164 µg/g). These results are consistent with the literature describing bees as efficient bioindicators of heavy metal pollution [48,49].

From a physiological perspective, Pb accumulation can be explained by ionic competition with Ca^2+^ and Zn^2+^ at the intestinal level, facilitating its transfer into the hemolymph and subsequently into venom-producing glands. The high levels during the acacia period suggest increased Pb bioavailability in local vegetation, possibly associated with traffic, agricultural activities, or atmospheric deposition [50].

Cadmium (Cd) was detected almost exclusively during the artificial feeding period (0.303–3.971 µg/g), being absent in samples from natural foraging. This distribution indicates a point source, likely related to sugar syrup or hive conditions at the beginning of the season. Physiologically, Cd accumulation may be influenced by the metabolic state of bees after wintering. Variants with probiotics showed slightly reduced values, which is consistent with the ability of lactic acid bacteria to adsorb Cd and reduce its bioavailability [51,52].

Chromium (Cr) showed moderate fluctuations (0–~6 µg/g), with values of 1.2–2.7 µg/g in April, moderate levels during the rapeseed period (1.5–3.4 µg/g; Rnp4: 1.573 ± 0.180 µg/g), and maximum values during the acacia period (up to 5.980 ± 0.322 µg/g in Anp8). In July, Cr was present in all samples (2–4.6 µg/g; Snp3: 4.514 ± 0.408 µg/g). These values suggest combined exposure to natural and anthropogenic sources, consistent with literature indicating the presence of Cr in fertilizers and pesticides [38]. Cr absorption mechanisms involve non-specific ion transporters, which may explain the pronounced differences observed during periods of intense metabolic activity [53].

The administered supplements did not constitute a direct source of contamination; however, some differences between variants suggest indirect physiological effects. Essential oils (V2–V4) were associated with greater differences in Pb levels during natural foraging periods, possibly by influencing detoxification enzymes [54]. In contrast, probiotics (V5–V7) were associated with lower Cd and, in some cases, Pb values, which may reflect the ability of microorganisms to bind metals in the intestinal lumen [40]. The combined probiotic + oregano variant (V8) showed moderate Pb and Cd levels but some of the highest Cr concentrations, suggesting complex interactions between metal metabolism and the bioactive compounds of supplements.

From an ecological perspective, the distribution of heavy metals in bee venom provides valuable information about environmental quality in foraging areas. High Pb and Cr levels during the acacia period indicate possible local pollution sources, while the absence of Cd in natural foraging suggests localized contamination limited to artificial feeding. The results support the use of venom as a sensitive biological matrix for pollution monitoring, complementary to honey and pollen, for which extensive data already exist [55].

### 2.8. Cluster Analysis of Physico-Chemical and Mineral Parameters

**Figure 3:**
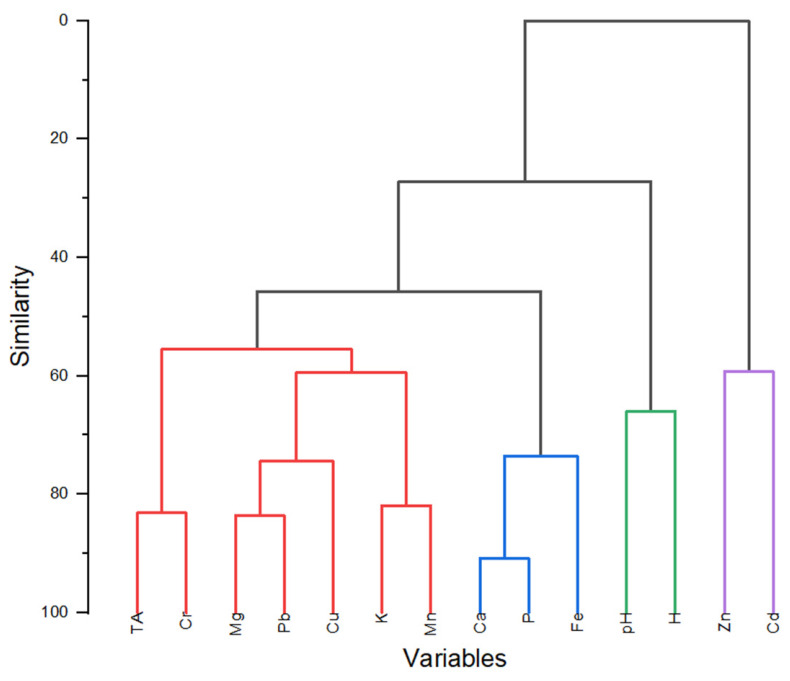
Hierarchical clustering dendrogram illustrating the similarity relationships among bee venom samples based on their physico-chemical and mineral parameters.

The dendrogram (Figure 3) highlights the grouping of variables according to the similarity of their variation in venom samples, outlining four distinct clusters with biological and ecological relevance. The largest cluster brings together total amino acids along with Mg, K, Mn, Cu, Cr, and Pb, indicating a common response to seasonal factors and floral sources. The simultaneous association of essential elements with certain toxic metals suggests that accumulation mechanisms are primarily influenced by trophic resources and environmental conditions, which corresponds to the pronounced variations observed during the acacia foraging period.

A second cluster, composed of Ca, P, and Fe, reflects a high correlation among these elements and a more stable behavior, characteristic of physiologically controlled variables. These elements are involved in structural and enzymatic processes, and their moderate and coordinated variations indicate stricter internal regulation compared to the macroelements in the first cluster.

The cluster grouping pH and moisture (H) highlights the association between parameters describing the chemical environment and the degree of venom dehydration. Their grouping suggests that variations in these parameters are determined by harvesting conditions and nectar characteristics, yet they remain relatively stable with respect to nutritional supplementation, confirming physiological control over venom acidity.

The final cluster, consisting of Zn and Cd, indicates a similar behavior of these metals, distinct from that of nutritional elements. Their grouping suggests common bioaccumulation mechanisms and a possible link to environmental contaminant exposure, a characteristic feature of elements with toxic potential.

The dendrogram confirms that the compositional variability of venom is predominantly driven by seasonal and ecological factors, while nutritional supplements exert limited and unsystematic effects. The clear separation of certain toxic metals, such as Zn and Cd, supports the potential of bee venom as a tool for biomonitoring environmental contamination.

**Figure 4:**
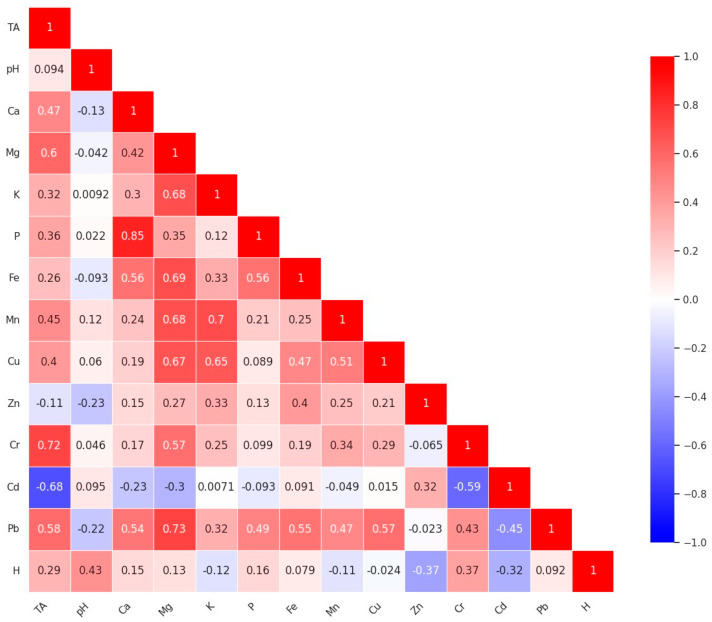
Pearson correlation matrix illustrating the relationships among environmental variables (temperature, humidity), hive-weight dynamics, and the physico-chemical and mineral parameters of bee venom.

The Pearson correlation (Figure 4) analysis reveals coherent groups of variables with similar patterns of variation. Total amino acids show moderate to strong positive correlations with several essential elements, including Ca (r = 0.47), K (r = 0.32), P (r = 0.36), Fe (r = 0.45), Mn (r = 0.45) and especially Cr (r = 0.72), indicating a common response to seasonal dynamics and the nutritional quality of floral resources. Ca, P and Fe form a tightly connected physiological core, reflected in strong correlations such as Ca–P (r = 0.85) and Ca–Fe (r = 0.58), suggesting coordinated regulation in structural and enzymatic processes.

pH correlates strongly with moisture (r = 0.52), highlighting the influence of harvesting conditions and nectar characteristics on the chemical environment of the venom while showing only weak associations with most minerals. Essential macro- and microelements also display notable interdependencies, including Mg–K (r = 0.68) and Mg–Mn (r = 0.67), consistent with their joint involvement in metabolic pathways and venom secretion.

Toxic metals exhibit distinct behaviors. Zn and Cu correlate positively with essential elements (e.g., Zn–Ca: r = 0.67; Cu–K: r = 0.51), suggesting similar uptake pathways through floral resources. In contrast, Cd shows strong negative correlations, particularly with TA (r = −0.68) and P (r = −0.59), indicating a divergent pattern likely linked to environmental contamination or detoxification responses. Pb displays moderate positive correlations with several variables (e.g., Pb–Mg: r = 0.37; Pb–K: r = 0.45), suggesting co-accumulation with essential minerals under certain environmental conditions.

The correlation matrix confirms the presence of distinct groups of elements with shared behavior and a clear separation of toxic metals, supporting the role of bee venom as a sensitive indicator of environmental exposure. The structure of correlations reinforces that seasonal and ecological factors are the primary drivers of compositional variability, whereas nutritional supplementation exerts only limited and inconsistent effects.

### 2.9. Seasonal Dynamics of Hive Weight, Temperature, and Humidity

**Figure 5:**
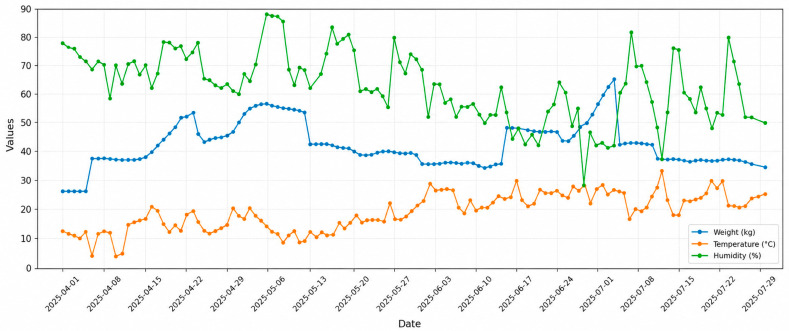
Evolution of weight, temperature, and humidity recorded in April–July 2025.

The pattern of hive-weight fluctuations from April to July (Figure 5) reflects how colony metabolism responds to changing climate, forage availability, and seasonal physiology. The relative stability recorded in April, when temperatures were still low and colonies relied on artificial feeding, corresponds to reduced foraging activity and slow population growth typical of early spring.

In May, although weather conditions improved, hive weight declined after an initial rise, indicating that nectar availability—not temperature—was the main limiting factor. This pattern reinforces the central role of natural forage in sustaining colony development and productivity.

June showed the most pronounced increase in hive weight, driven by optimal temperature–humidity conditions and the abundance of acacia nectar. This period corresponds to peak foraging intensity and elevated metabolic activity, conditions that also favor the synthesis of bioactive compounds in bee venom. By contrast, July was characterized by a gradual decline in hive weight despite high temperatures, reflecting reduced floral resources and increased internal consumption.

These seasonal dynamics align with findings by Gunasekara et al. (2024) [56], who demonstrated that ecological and seasonal conditions strongly influence venom composition. Isidorov et al. (2023) [3] similarly emphasized the combined effects of nutrition and environment on metabolic activity and secretory processes in bees. Zidan et al. (2026) [57] further confirmed that both diet type and season shape colony development and venom production while highlighting the sensitivity of venom composition to floral origin, environmental conditions, and colony physiology.

Taken together, the findings show that nectar availability is the dominant factor shaping hive–weight dynamics, while temperature and humidity act as secondary modulators that influence foraging efficiency and the metabolic processes underpinning venom biosynthesis. In parallel, these seasonal shifts in hive weight, temperature, and humidity mirror the variations observed in the elemental and mineral composition of bee venom, emphasizing how environmental conditions simultaneously regulate colony physiology and the chemical profile of venom.

Although temperature and humidity were not included in multivariate statistical analyses, their influence on venom composition was assessed in relation to colony foraging activity, metabolic intensity, and biochemical processes involved in venom biosynthesis. Because meteorological data were recorded at the hive level and not at the level of individual venom samples, the number of observations per sampling period was insufficient for robust statistical models or correlation analyses. For this reason, temperature and humidity were used as ecological context variables and not as independent predictors in statistical tests.

Even in the absence of formal statistical integration, seasonal patterns of temperature, humidity, and hive weight dynamics provide relevant information on how environmental conditions regulate colony physiology. Periods characterized by an optimal temperature–humidity balance coincided with an intensification of foraging activity and metabolism, conditions also favorable for the synthesis of bioactive compounds in venom. In contrast, periods of reduced nectar availability—even under high temperatures—were reflected in reduced hive weight and were associated with changes in venom composition. These ecological relationships support the hypothesis that meteorological factors indirectly influence venom quality by shaping foraging behavior, resource intake, and the metabolic state of the colony.

## 3. Materials and Methods

### 3.1. Collection of Venom Samples

For this study, a total of 32 bee venom samples obtained from *Apis mellifera carpatica* colonies were analyzed, collected in four successive stages: April, May, June, and July 2025. The apiary was located in Timiș County, Romania. For statistical validation, three bee colonies were selected for each administered supplement.

**Figure 6:**
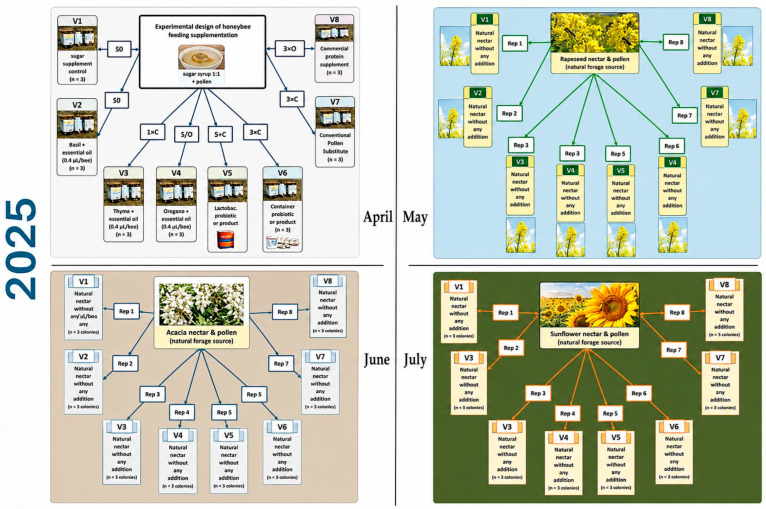
Experimental design.

The first harvest was carried out after a three-week period of stimulative feeding with sugar syrup (1:1 ratio), supplemented with essential oils (thyme, oregano, basil) or commercial probiotic products. Table 3 and Figure 6 present the complete experimental design, including the type of supplement used and the venom sample code.

Within the experiment, natural essential oils purchased from the Romanian producer Bioinovativ (https://life-bio.ro—accessed on 11 March 2026), certified by the National Service for Medicinal and Aromatic Plants and Beekeeping Products, were used. These oils were obtained by steam distillation and included whole thyme essential oil (*Thymus vulgaris*), whole wild oregano essential oil (*Origanum vulgare*), and whole basil essential oil (*Ocimum basilicum*). All oils were used at a dose of 30 µL per colony, added to sugar syrup, according to methodologies previously applied by other researchers [45].

For probiotic supplementation, the following commercial products were used:

- Lacium—contains 50 billion CFU (colony-forming units) per capsule, composed of 9 strains of lactobacilli and bifidobacteria: *Bifidobacterium breve* BR03, *Lactobacillus rhamnosus* LR02, *Lactobacillus plantarum* LP09, *Lactobacillus casei* LC03, *Lactobacillus acidophilus* LA02, *Streptococcus thermophilus* YA08, *Bifidobacterium bifidum* BB01, *Bifidobacterium longum*, and *Lactococcus lactis* LL02. Each capsule contains 162 mg of probiotic strains and 140 mg of inulin.
- Colobiotic—contains 8 strains of beneficial microorganisms (200 mg per capsule), including *Bacillus coagulans*, *Bacillus subtilis natto*, *Lactobacillus acidophilus*, *Lactobacillus casei*, *Lactobacillus rhamnosus*, *Lactobacillus plantarum*, *Bifidobacterium bifidum*, and *Bifidobacterium breve*.
- Enterolactis Plus—a single-component product containing 250 mg/capsule of *Lactobacillus paracasei* CNCM I-1572, known for its ability to resist gastric acidity and to colonize the intestinal tract.

The second sample collection was performed after the rapeseed flowering period (*Brassica napus*), the third after the acacia harvest (*Robinia pseudoacacia*), and the fourth after the sunflower harvest (*Helianthus annuus*), without syrup supplementation, in order to allow comparison with natural food sources.

**Figure 7:**
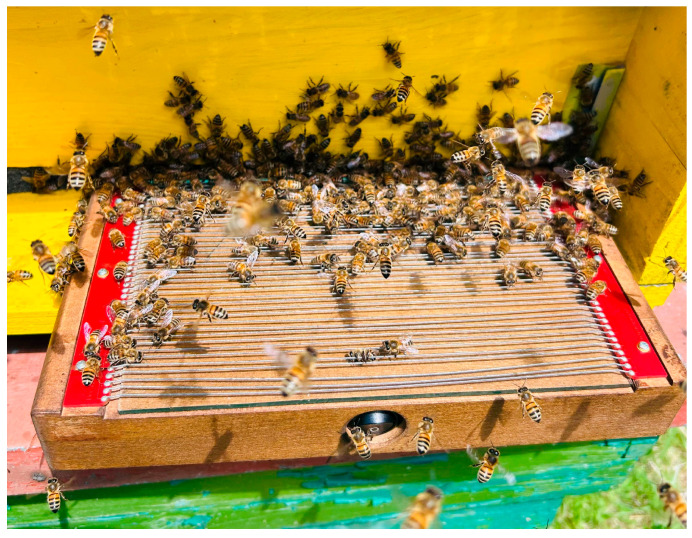
BeeWhisper v. 5.1 collector model 2016.

Venom collection was carried out using the BeeWhisper v.5.1 collector, 2016 model (Figure 7), with a stimulation duration of 30 min per colony, using an electrical stimulation method described in other studies and research as well [45].

### 3.2. Determination of Moisture Content and Dry Matter

Moisture content was determined using a precision thermobalance (POL-EKO APARATURA laboratory oven, Poland), according to the standard drying method at controlled temperatures (100–105 °C). Samples of approximately 0.01 g were weighed and analyzed individually until mass stabilization. The obtained values were expressed as percentages (%), and dry matter content was calculated indirectly by difference (100 − moisture) [45].

### 3.3. Determination of pH

The pH value was measured using the conductometric method with a digital pH meter (Mettler Toledo SevenEasy). For each determination, 0.003 g of venom was dissolved in 10 mL of distilled water and homogenized for 30 min [45,58,59] using a magnetic stirrer (DLAB SK-L330-PRO).

### 3.4. Determination of Ash Content

Total ash content was determined by the loss-on-ignition method, using a Nabertherm furnace (POL-EKO APARATURA laboratory oven, Wodzisław Śląski Poland). Samples of 0.1 g were incinerated at 525 °C for 2 h until a white–gray ash was obtained. Calculation formula: the ash content (C_ash_) is equal to the ratio between the difference in the mass of the crucible with ash and the mass of the empty crucible and the difference between the mass of the crucible with the sample before incineration and the mass of the empty crucible, multiplied by 100 [45].

C_ash_ = (m − m1)/(m2 − m1) (%) (1)

where m = mass of the crucible with ash (g), m_1_ = mass of the empty crucible (g), and m_2_ = mass of the crucible with the sample before incineration (g).

### 3.5. Determination of Total Amino Acids

The total amino acid content was determined using the ninhydrin colorimetric method after acid hydrolysis, according to the procedure described in [45], with minor modifications. 3 mg of dried bee venom was transferred to hydrolysis tubes and treated with 5 mL of 6 N HCl. The tubes were flushed with nitrogen to prevent oxidative degradation, sealed, and incubated at 110 °C for 24 h in a thermostated oven. After hydrolysis, the samples were cooled to room temperature, filtered, and excess hydrochloric acid was removed by drying at ≤40 °C. The residue was subsequently reconstituted in a defined volume of distilled water. An aliquot of the hydrolyzed extract was treated with phosphate buffer (pH 8.0) and 2% (*w*/*v*) ninhydrin reagent, followed by heating at 100–103 °C for color development. After cooling, the absorbance was measured at 570 nm using a UV-VIS spectrophotometer (Analytik Jena Specord 210 Plus, Jena, Germany). Quantification was performed using a calibration curve prepared with alanine standard solutions (100–600 µg/mL).

### 3.6. Mineral Content

#### 3.6.1. Determination of Total Metal Content

Bee venom samples were processed for metal determination using a dry ashing procedure. Initially, the samples were placed in open crucibles and heated in a muffle furnace at 550 °C under atmospheric pressure, allowing the organic components to be thermally decomposed and eliminated.

The ash obtained after this process was dissolved in 6 N hydrochloric acid (HCl). The solution was subsequently filtered and diluted with bidistilled water to a final volume of 50 mL.

Metal concentrations were then measured by atomic absorption spectrometry (AAS) using a Varian Spectra 240 FS spectrophotometer (Palo Alto, CA, USA). The instrumental parameters applied during the analysis are presented in Table 4.

During analysis, an air–acetylene flame with a ratio of 13.50:2 was used, while the nebulizer uptake rate was set to 5 L/min.

Calibration curves were established using standard solutions with concentrations between 0.3 and 3 µg/L, prepared from a multi-element ICP standard solution (1000 mg/L).

Although Ni was included in the mineral analysis, all measurements were below the detection limit of the ICP OES method and therefore could not be reported.

#### 3.6.2. Determination of Total Phosphorus

Phosphorus content was evaluated spectrophotometrically using the ammonium phosphomolybdate method, which reacted with stannous chloride to form a colored complex. Absorbance was measured at 715 nm using a UV–VIS spectrophotometer, Analytik Jena Specord 210 Plus (Jena, Germany), with standard phosphorus solutions of 2–60 µg/100 mL.

### 3.7. Monitoring of Meteorological Conditions and Colony Activity

**Figure 8:**
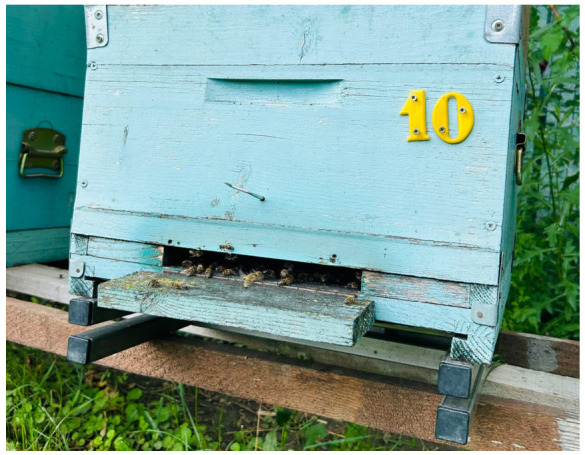
BeeWatch Professional.

To evaluate the influence of environmental factors on the quantity and quality of bee venom, the BeeWatch Professional monitoring system (Figure 8) was used. This system was installed beneath a multi-story hive representative of the average colony conditions in the apiary and remained operational throughout the entire experimental period (April–July 2025).

BeeWatch Professional provides real-time, high-precision data on ambient temperature (°C), relative air humidity (%), and changes in the total weight of the hive, reflecting feed consumption and the activity level of the colony.

Monitoring was carried out continuously, on an hourly basis, across four distinct experimental stages: the period of artificial stimulative feeding, the rapeseed foraging period (*Brassica napus*), the acacia foraging period (*Robinia pseudoacacia*), and the sunflower foraging period (*Helianthus annuus*).

By correlating the evolution of meteorological factors with the monitored biological parameters (colony development, feed consumption, and venom production), it was possible to assess the indirect impact of climatic variations on the secretion and quality of bee venom. In particular, sudden fluctuations in temperature and humidity were analyzed in relation to behavioral changes and the efficiency of supplemental feeding.

This approach allows for a more comprehensive understanding of the interaction between external factors and the physiological response of bee colonies under real field conditions, confirming the importance of modern monitoring technologies in contemporary apicultural research.

### 3.8. Statistical Analysis

All data were statistically analyzed using OriginPro 2025 software (OriginLab Corporation, Northampton, MA, USA), and significant differences between means (*p* < 0.05) were tested by ANOVA followed by Tukey’s post hoc test. Pearson correlation analysis and hierarchical cluster analysis (dendrogram) were also performed.

## 4. Conclusions

The present study shows that bee venom is a highly dynamic biological product whose composition is shaped predominantly by seasonal conditions and botanical origin, with nutritional supplementation exerting only secondary effects. The physico-chemical parameters—moisture, dry matter, pH, and ash—remained within the characteristic ranges reported for bee venom, indicating strong physiological regulation. Among these, pH proved the most stable, whereas moisture and dry matter responded more visibly to environmental and seasonal variation.

Total amino acid content varied markedly with floral source, reaching its highest levels during the acacia foraging period. This pattern underscores the essential role of natural nutrition in supporting protein synthesis and enhancing venom quality, while artificial supplementation did not consistently improve total amino acid levels.

The macroelement profile was dominated by potassium, followed by calcium, phosphorus, and magnesium, with clear seasonal trends. Acacia nectar again produced the highest concentrations, confirming botanical origin as the primary determinant of mineral composition. Microelements such as Fe, Mn, Cu, and Zn showed more complex patterns influenced by both diet and environmental exposure, though natural forage remained the strongest and most consistent driver.

Heavy metal analysis highlighted the value of bee venom as an environmental bio-indicator. Elevated levels of lead and chromium during natural foraging—particularly in the acacia season—reflected environmental contamination, while cadmium was more closely associated with artificial feeding conditions. These findings reinforce the sensitivity of venom to local ecological conditions.

Multivariate analyses supported these conclusions, showing that seasonal and ecological factors cluster as the dominant influences on venom composition, whereas supplements produced limited and inconsistent effects. The grouping of essential minerals with certain toxic metals further suggests shared environmental pathways of uptake.

Overall, the results indicate that high-quality bee venom depends primarily on access to diverse, abundant, and seasonally appropriate floral resources. While supplements may adjust specific parameters, they cannot replicate the complex nutritional and ecological contributions of natural forage. Future work should integrate proteomic, metabolomic, and environmental monitoring approaches to clarify the mechanisms driving venom variability and to support the standardization, safety, and optimization of venom harvesting for apitherapeutic use.

## Figures and Tables

**Table 1:** Moisture content, dry matter, pH, ash and total amino acids of bee venom.

Variant		Moisture (%)	DM (%)	pH	Ash (%)	Total Amino Acids (mg/g)
V1	Ss	14.8 ± 0.2 ^abcB^	85.2 ± 0.2 ^abcB^	6.25 ± 0.05 ^aA^	4.2 ± 0.2 ^cdAB^	385.5 ± 15.8 ^aB^
	Rnp1	14.8 ± 0.5 ^cB^	85.2 ± 0.5 ^cB^	6.15 ± 0.05 ^aA^	4.8 ± 0.3 ^aA^	380.3 ± 2.4 ^cB^
	Anp1	14.2 ± 0.6 ^cB^	85.8 ± 0.6 ^cB^	6.03 ± 0.03 ^aB^	4.7 ± 0.3 ^aA^	409.5 ± 0.6 ^aA^
	Snp1	16.8 ± 0.9 ^bA^	83.2 ± 0.9 ^bA^	6.12 ± 0.04 ^aB^	3.5 ± 0.4 ^aB^	396.4 ± 1 ^aAB^
V2	SsB	14.5 ± 0.4 ^cdC^	85.9 ± 0.4 ^cdC^	5.89 ± 0.04 ^cdB^	5.2 ± 0.3 ^abA^	354.5 ± 15.9 ^bB^
	Rnp2	19.2 ± 0.5 ^aA^	80.8 ± 0.5 ^aA^	6.04 ± 0.04 ^aA^	3.1 ± 0.3 ^dC^	345.5 ± 5.8 ^eB^
	Anp2	14.9 ± 0.4 ^cC^	85.1 ± 0.4 ^cC^	6.09 ± 0.03 ^aA^	4.3 ± 0.4 ^abAB^	403.2 ± 2.9 ^bcA^
	Snp2	17.2 ± 0.4 ^abB^	82.8 ± 0.4 ^abB^	6.14 ± 0.05 ^aA^	4.2 ± 0.4 ^aB^	387.1 ± 2.9 ^cdA^
V3	SsT	14.6 ± 0.3 ^abcC^	85.1 ± 0.3 ^abcC^	6.14 ± 0.04 ^abA^	4.6 ± 0.3 ^bcA^	344 ± 7.5 ^bC^
	Rnp3	16.3 ± 0.5 ^bB^	83.7 ± 0.5 ^bB^	6.06 ± 0.04 ^aA^	3.9 ± 0.4 ^cdA^	360.9 ± 5.1 ^dB^
	Anp3	16.5 ± 0.7 ^bB^	83.5 ± 0.7 ^bB^	5.97 ± 0.03 ^bB^	4.2 ± 0.4 ^abcA^	400.4 ± 2.5 ^cA^
	Snp3	18.4 ± 0.4 ^aA^	81.6 ± 0.4 ^aA^	6.02 ± 0.06 ^aB^	3.9 ± 0.3 ^aA^	388.5 ± 1.1 ^bcdA^
V4	SsO	12.8 ± 0.5 ^dB^	86.7 ± 0.5 ^dB^	5.97 ± 0.03 ^bcB^	5.5 ± 0.4 ^aA^	364.6 ± 4.8 ^abD^
	Rnp4	16.6 ± 0.4 ^bA^	83.4 ± 0.4 ^bA^	5.88 ± 0.04 ^bB^	3.6 ± 0.1 ^cdB^	378.6 ± 3.9 ^cC^
	Anp4	17 ± 0.5 ^abA^	83 ± 0.5 ^abA^	6.04 ± 0.03 ^aA^	4.1 ± 0.3 ^abcdB^	405.6 ± 2.6 ^abcA^
	Snp4	17.6 ± 0.5 ^abA^	82.4 ± 0.5 ^abA^	6.07 ± 0.04 ^aA^	4.4 ± 0.5 ^aB^	391.2 ± 0.8 ^bcB^
V5	SsL	16.1 ± 0.5 ^abB^	84.4 ± 0.5 ^abB^	6.02 ± 0.05 ^bcA^	3.8 ± 0.2 ^cdB^	362 ± 8.1 ^abC^
	Rnp5	13.8 ± 0.3 ^cC^	86.2 ± 0.3 ^cC^	5.81 ± 0.06 ^cB^	5 ± 0.3 ^aA^	390.4 ± 0.6 ^bB^
	Anp5	17.2 ± 0.7 ^abA^	82.8 ± 0.7 ^abA^	6.02 ± 0.08 ^aA^	3.7 ± 0.1 ^bcdB^	406.9 ± 1.2 ^abA^
	Snp5	17.2 ± 0.6 ^abA^	82.8 ± 0.6 ^abA^	6.11 ± 0.06 ^aA^	4.5 ± 0.3 ^aA^	379.5 ± 1.6 ^eB^
V6	SsC	15.5 ± 0.4 ^aB^	84.1 ± 0.4 ^aB^	5.8 ± 0.05 ^dB^	3.5 ± 0.3 ^dB^	368.8 ± 7.9 ^abD^
	Rnp6	11.2 ± 0.3 ^dC^	88.8 ± 0.3 ^dC^	5.6 ± 0.06 ^dC^	5.3 ± 0.2 ^aAB^	377.8 ± 2.2 ^bB^
	Anp6	18 ± 0.2 ^aA^	82 ± 0.2 ^aA^	6.1 ± 0.05 ^aA^	3.5 ± 0.4 ^bcdB^	407 ± 1.6 ^abA^
	Snp6	18 ± 0.5 ^abA^	82 ± 0.5 ^abA^	6.09 ± 0.04 ^aA^	4 ± 0.2 ^aB^	384.2 ± 1.1 ^deC^
V7	SsE	14.3 ± 0.7 ^abcC^	85 ± 0.7 ^abcC^	5.97 ± 0.03 ^bcA^	3.9 ± 0.2 ^cdAB^	370.5 ± 6.5 ^abD^
	Rnp7	16.5 ± 0.5 ^bB^	83.5 ± 0.5 ^bB^	5.92 ± 0.04 ^bA^	4 ± 0.4 ^bcAB^	394.7 ± 2.3 ^bB^
	Anp7	18.4 ± 0.3 ^aA^	81.6 ± 0.3 ^aA^	5.89 ± 0.03 ^bA^	3.3 ± 0.3 ^cdB^	406.7 ± 0.9 ^abA^
	Snp7	17.9 ± 0.5 ^abA^	82.1 ± 0.5 ^abA^	6.04 ± 0.95 ^aA^	4.3 ± 0.4 ^aA^	388.9 ± 2.3 ^bcdC^
V8	SsPO	14.3 ± 0.3 ^bcB^	85.4 ± 0.3 ^bcB^	6.03 ± 0.04 ^dB^	4.3 ± 0.4 ^cdA^	361.6 ± 3.8 ^abC^
	Rnp8	16.9 ± 0.5 ^bA^	83.1 ± 0.5 ^bA^	6 ± 0.04 ^bB^	3.8 ± 0.2 ^cdAB^	408.5 ± 1.6 ^aA^
	Anp8	18 ± 0.6 ^aA^	82 ± 0.6 ^aA^	6.09 ± 0.04 ^aA^	3.2 ± 0.3 ^dB^	408.1 ± 0.9 ^abA^
	Snp8	18.1 ± 0.4 ^abA^	81.9 ± 0.4 ^abA^	6.14 ± 0.04 ^aA^	3.6 ± 0.4 ^aAB^	393.1 ± 1.5 ^abB^

Means marked with the same letters show no statistically significant differences (*p* > 0.05), while means associated with different letters demonstrate statistically significant differences (*p* < 0.05). Lowercase letters are used to denote statistically significant differences between groups within the same harvest, whereas uppercase letters indicate statistically significant differences between harvests.

**Table 2:** Macroelement content of bee venom.

Variant		Ca (mg/g)	K (mg/g)	Mg (mg/g)	P (mg/g)
V1	Ss	1.809 ± 0.341 ^aB^	6.312 ± 0.335 ^bcC^	0.171 ± 0.011 ^cD^	1.120 ± 0.064 ^aA^
	Rnp1	1.224 ± 0.340 ^deC^	7.942 ± 0.336 ^cB^	0.333 ± 0.019 ^bB^	0.543 ± 0.039 ^bcC^
	Anp1	2.526 ± 0.17 ^dA^	8.170 ± 0.470 ^aA^	0.951 ± 0.161 ^bA^	0.921 ± 0.025 ^bcB^
	Snp1	1.486 ± 0.248 ^eD^	3.973 ± 0.203 ^aD^	0.241 ± 0.024 ^bC^	0.354 ± 0.064 ^aD^
V2	SsB	1.618 ± 0.383 ^fD^	4.425 ± 0.285 ^eBC^	0.106 ± 0.012 ^dB^	0.518 ± 0.029 ^dB^
	Rnp2	1.245 ± 0.289 ^deB^	4.621 ± 0.331 ^fgB^	0.251 ± 0.011 ^eB^	0.539 ± 0.037 ^bcB^
	Anp2	3.686 ± 0.276^bA^	10.341 ± 0.747^bA^	0.456 ± 0.065^dA^	1.848 ± 0.154^bcA^
	Snp2	1.769 ± 0.303 ^cC^	3.320 ± 0.431 ^bC^	0.775 ± 0.026 ^fC^	0.402 ± 0.046 ^aB^
V3	SsT	1.207 ± 0.305 ^dC^	6.839 ± 0.209 ^abB^	0.206 ± 0.012 ^bB^	0.843 ± 0.040 ^bcB^
	Rnp3	1.262 ± 0.143 ^deC^	11.064 ± 0.282 ^bA^	0.194 ± 0.052 ^dB^	0.606 ± 0.025 ^abC^
	Anp3	3.400 ± 0.284 ^cA^	11.059 ± 0.497 ^bA^	0.298 ± 0.039 ^bA^	1.531 ± 0.035 ^bcA^
	Snp3	2.650 ± 0.276 ^aB^	3.759 ± 0.045 ^bC^	0.127 ± 0.017 ^dC^	0.551 ± 0.049 ^aC^
V4	SsO	1.707 ± 0.376 ^bA^	5.814 ± 0.369 ^cdB^	0.265 ± 0.075 ^aB^	1.043 ± 0.060 ^aA^
	Rnp4	1.126 ± 0.233 ^efB^	5.635 ± 0.315 ^deBC^	0.793 ± 0.040 ^fC^	0.509 ± 0.016 ^cC^
	Anp4	1.005 ± 0.129 ^eC^	4.939 ± 0.033 ^dC^	0.575 ± 0.020 ^cA^	0.839 ± 0.039 ^cB^
	Snp4	1.519 ± 0.158 ^deD^	9.137 ± 0.192 ^dA^	0.550 ± 0.014 ^aA^	0.368 ± 0.07 ^aD^
V5	SsL	0.830 ± 0.021 ^eC^	4.994 ± 0.419 ^deB^	0.117 ± 0.038 ^dC^	0.731 ± 0.071 ^cA^
	Rnp5	2.727 ± 0.312 ^bA^	6.369 ± 0.197 ^deB^	0.244 ± 0.076 ^cB^	0.634 ± 0.037 ^abA^
	Anp5	1.062 ± 0.640 ^eB^	7.043 ± 0.434 ^cA^	0.560 ± 0.010 ^cA^	0.644 ± 0.032 ^cA^
	Snp5	1.626 ± 0.345 ^deD^	4.955 ± 0.253 ^cB^	0.101 ± 0.027 ^efD^	0.373 ± 0.018 ^aB^
V6	SsC	1.376 ± 0.215 ^cB^	7.690 ± 0.438 ^abA^	0.194 ± 0.052 ^bA^	0.898 ± 0.044 ^bB^
	Rnp6	1.057 ± 0.138 ^fC^	5.091 ± 0.284 ^efB^	0.207 ± 0.082 ^dA^	0.412 ± 0.040 ^dC^
	Anp6	1.648 ± 0.167 ^aA^	5.170 ± 0.290 ^dC^	0.193 ± 0.053 ^eA^	0.421 ± 0.021 ^aA^
	Snp6	2.083 ± 0.102 ^fD^	3.967 ± 0.427 ^dD^	0.124 ± 0.014 ^deB^	0.319 ± 0.017 ^aC^
V7	SsE	0.861 ± 0.011 ^eC^	4.820 ± 0.198 ^eC^	0.125 ± 0.025 ^dC^	0.802 ± 0.016 ^bcB^
	Rnp7	1.473 ± 0.277 ^aB^	3.195 ± 0.251 ^aA^	0.449 ± 0.015 ^aB^	0.695 ± 0.026 ^aB^
	Anp7	1.651 ± 0.270 ^aA^	7.097 ± 0.226 ^cB^	0.106 ± 0.035 ^aA^	0.314 ± 0.012 ^abA^
	Snp7	0.806 ± 0.143 ^cC^	4.487 ± 0.453 ^cC^	0.148 ± 0.026 ^dC^	0.448 ± 0.034 ^aC^
V8	SsPO	1.495 ± 0.194 ^gD^	3.445 ± 0.211 ^fC^	0.671 ± 0.051 ^eC^	0.351 ± 0.037 ^eD^
	Rnp8	2.575 ± 0.167 ^cA^	3.885 ± 0.203 ^gC^	0.196 ± 0.010 ^dB^	0.618 ± 0.043 ^abB^
	Anp8	1.075 ± 0.295 ^eC^	11.372 ± 0.405 ^bA^	0.100 ± 0.038 ^abA^	0.752 ± 0.031 ^cA^
	Snp8	1.498 ± 0.118 ^bB^	7.282 ± 0.312 ^bB^	0.208 ± 0.010 ^cB^	0.508 ± 0.031 ^aC^

Means marked with the same letters show no statistically significant differences (*p* > 0.05), while means associated with different letters demonstrate statistically significant differences (*p* < 0.05). Lowercase letters are used to denote statistically significant differences between groups within the same harvest, whereas uppercase letters indicate statistically significant differences between harvests.

**Table 3:** Detailed experimental design.

Sample No.	Harvest Period	Sample Code	Second Sample Code	Supplement Type	Volume/Dosage	Type of Basic Feed	Number of Colonies
1	April	V1	Ss	No supplement	1 L	Sugar syrup 1:1	3
2	April	V2	SsB	Basil essential oil	1 L + 30 µL	Sugar syrup 1:1	3
3	April	V3	SsT	Thyme essential oil	1 L + 30 µL	Sugar syrup 1:1	3
4	April	V4	SsO	Oregano essential oil	1 L + 30 µL	Sugar syrup 1:1	3
5	April	V5	SsL	Lacium probiotic	1 L + 1 capsule	Sugar syrup 1:1	3
6	April	V6	SsC	Colobiotic probiotic	1 L + 1 capsule	Sugar syrup 1:1	3
7	April	V7	SsE	Enterolactis Plus probiotic	1 L + 1 capsule	Sugar syrup 1:1	3
8	April	V8	SsPO	Probiotic + oregano	1 L + 30 µL + 1 capsule	Sugar syrup 1:1	3
9	May	V1	Rnp1	No supplement	-	Rapeseed nectar and pollen	3
10	May	V2	Rnp2	No supplement	-	Rapeseed nectar and pollen	3
11	May	V3	Rnp3	No supplement	-	Rapeseed nectar and pollen	3
12	May	V4	Rnp4	No supplement	-	Rapeseed nectar and pollen	3
13	May	V5	Rnp5	No supplement	-	Rapeseed nectar and pollen	3
14	May	V6	Rnp6	No supplement	-	Rapeseed nectar and pollen	3
15	May	V7	Rnp7	No supplement	-	Rapeseed nectar and pollen	3
16	May	V8	Rnp8	No supplement	-	Rapeseed nectar and pollen	3
17	June	V1	Anp1	No supplement	-	Acacia nectar and pollen	3
18	June	V2	Anp2	No supplement	-	Acacia nectar and pollen	3
19	June	V3	Anp3	No supplement	-	Acacia nectar and pollen	3
20	June	V4	Anp4	No supplement	-	Acacia nectar and pollen	3
21	June	V5	Anp5	No supplement	-	Acacia nectar and pollen	3
22	June	V6	Anp6	No supplement	-	Acacia nectar and pollen	3
23	June	V7	Anp7	No supplement	-	Acacia nectar and pollen	3
24	June	V8	Anp8	No supplement	-	Acacia nectar and pollen	3
25	July	V1	Snp1	No supplement	-	Sunflower nectar and pollen	3
26	July	V2	Snp2	No supplement	-	Sunflower nectar and pollen	3
27	July	V3	Snp3	No supplement	-	Sunflower nectar and pollen	3
28	July	V4	Snp4	No supplement	-	Sunflower nectar and pollen	3
29	July	V5	Snp5	No supplement	-	Sunflower nectar and pollen	3
30	July	V6	Snp6	No supplement	-	Sunflower nectar and pollen	3
31	July	V7	Snp7	No supplement	-	Sunflower nectar and pollen	3
32	July	V8	Snp8	No supplement	-	Sunflower nectar and pollen	3

**Table 4:** Detailed Experimental Design.

Metal	Cr	Cu	Cd	Mn	Zn	Fe	Ca	Mg	Pb	K
λ (nm)	357.9	324.8	228.8	279.5	213	248.3	422.7	285.2	283.3	414
Lamp Current (mA)	7	4	4	5	5	5	10	4	10	4
Slit Width (mm)	0.2	0.5	0.5	0.2	1	0.2	0.5	0.5	1.2	0.5

## Data Availability

The original contributions presented in the study are included in the article. Further inquiries can be directed to the corresponding authors.

## Supplementary Materials

The following supporting information can be downloaded at: https://www.mdpi.com/article/10.3390/molecules31111834/s1, Table S1: Microelement content of bee venom; Table S2: Toxic metal content of bee venom.
