# Supplementary material for: Physico-Chemical and Mineral Variability of Apis mellifera Bee Venom Across Seasons and Feeding Regimes
Source: Molecules. 2026 May 26;31(11):1834. doi: 10.3390/molecules31111834 (PMC13258300; doi:10.3390/molecules31111834)
Supplement: Supplementary file 1 [file molecules-31-01834-s001.zip › molecules-4268198-supplementary.pdf]

Table S1. Microelement content of bee venom

Variant		Fe(mg/g)	Mn(μg/g)	Cu(μg/g)	Zn(mg/g)
V1	Ss	0.480 ± 0.023^{aA}	3.267 ± 0.190^{aB}	4.526 ± 0.356^{aB}	1.710 ± 0.111^{bcB}
	Rnp1	0.382± 0.024^{aB}	2.803 ± 0.250^{aB}	7.256 ± 0.409^{aA}	1.230 ± 0.429^{cdC}
	Anp1	0.505 ± 0.026^{cA}	5.050± 0.140^{bA}	6.941 ± 0.211^{bA}	2.640 ± 0.130^{aA}
	Snp1	0.981 ± 0.088^{cC}	1.207 ± 0.167^{bC}	1.951 ± 0.143^{cdC}	1.161 ± 0.250^{bcC}
V2	SsB	0.117 ± 0.016^{efB}	1.293 ± 0.254^{cdB}	1.974 ± 0.150^{efC}	2.333 ± 0.195^{abA}
	Rnp2	0.148 ± 0.025^{deB}	1.191 ± 0.128^{bB}	2.730 ± 0.123^{dB}	0.879 ±0.038^{eC}
	Anp2	0.282 ± 0.026^{deA}	6.066 ± 0.258^{bA}	4.245 ± 0.357^{cA}	2.150 ± 0.169^{abcA}
	Snp2	0.715 ± 0.033^{deC}	7.171 ± 0.126^{cdB}	1.451 ± 0.122^{cdC}	1.594 ± 0.085^{abB}
V3	SsT	0.500 ± 0.015^{aB}	1.604 ± 0.128^{cdB}	2.917 ± 0.356^{cdC}	2.883 ± 0.255^{aA}
	Rnp3	0.192 ± 0.038^{cC}	1.634 ± 0.133^{bB}	5.032 ± 0.184^{bB}	1.156 ± 0.087^{cdeC}
	Anp3	0.980 ± 0.0 18^{bA}	3.487 ± 0.142^{cA}	8.819 ± 0.372^{aA}	1.959 ± 0.165^{bcdB}
	Snp3	0.817 ± 0.064^{bD}	8.849 ± 0.206^{bcB}	1.619 ± 0.197^{cdD}	1.897 ± 0.314^{aB}
V4	SsO	0.292 ± 0.027^{cAB}	2.360 ± 0.140^{bAB}	4.022 ± 0.407^{abC}	2.905 ± 0.116^{aA}
	Rnp4	0.266 ± 0.013^{bB}	1.580 ± 0.125^{bC}	3.826 ± 0.121^{cC}	1.675 ± 0.314^{abB}
	Anp4	0.353 ± 0.028^{deA}	2.850 ± 0.217^{cdA}	8.610 ± 0.615^{aA}	1.817 ± 0.275^{bcdB}
	Snp4	0.235 ± 0.022^{deB}	1.709 ± 0.125^{aBC}	6.702 ± 0.226^{aB}	1.671 ± 0.143^{aB}
V5	SsL	0.186 ± 0.074^{dB}	1.197 ± 0.097^{cdB}	1.303 ± 0.442^{fC}	1.814 ± 0.203^{bcA}
	Rnp5	0.136 ± 0.033^{deBC}	1.504 ± 0.203^{bAB}	3.696 ± 0.149^{cB}	1.877 ± 0.091^{aA}
	Anp5	0.316 ± 0.087^{deA}	1.716 ± 0.189^{dA}	5.168 ± 0.726^{cA}	1.596 ± 0.210^{cdeA}
	Snp5	0.535 ± 0.042^{deC}	5.038 ± 0.250^{dC}	1.153 ± 0.138^{dC}	1.058 ± 0.136^{cB}
V6	SsC	0.429 ± 0.047^{bA}	2.274 ± 0.265^{bA}	3.388 ± 0.206^{bcA}	2.706 ± 0.256^{aA}
	Rnp6	0.115 ± 0.060^{eC}	1.624 ± 0.259^{bB}	2.432 ± 0.383^{dB}	1.493 ± 0.119^{bcBC}
	Anp6	0224 ± 0.017^{eB}	1.691 ± 0.114^{dB}	2.179 ± 0.315^{dB}	1.182 ± 0.069^{eC}
	Snp6	0.976 ± 0.080^{eC}	8.338 ± 0.581^{cdC}	2.292 ± 0.379^{cB}	1.865 ± 0.121^{aB}
V7	SsE	0.148 ± 0.077^{efBC}	1.254 ± 0.221^{cdC}	2.318 ± 0.106^{deC}	1.714 ± 0.392^{bcAB}
	Rnp7	0.188 ± 0.085^{cB}	2.789 ± 0.235^{aA}	5.675 ± 0.426^{bA}	1.913 ± 0.282^{aAB}
	Anp7	0.155 ± 0.067^{aAB}	1.922 ± 0.128^{dB}	4.118 ± 0.202^{cB}	2.243 ± 0.092^{abA}
	Snp7	0.849 ± 0.049^{aC}	8.343 ± 0.148^{cdC}	2.067 ± 0.151^{cC}	1.439 ± 0.146^{abcB}
V8	SsPO	0.978 ± 0.022^{fC}	6.900 ± 0.420^{dB}	1.225 ± 0.316^{fB}	1.194 ± 0.149^{cAB}
	Rnp8	0.153 ± 0.040^{dB}	1.774 ± 0.267^{bB}	2.479 ± 0.342^{dB}	1.049 ± 0.127^{deB}
	Anp8	0.205 ± 0.048^{eA}	7.854 ± 0.717^{aA}	4.246 ± 0.373^{cA}	1.501 ± 0.384^{deAB}
	Snp8	0.230 ± 0.047^{eA}	1.716 ± 0.133^{aB}	4.630 ± 0.548^{bA}	1.695 ± 0.154^{aA}

Means marked with the same letters show no statistically significant differences ($p > 0.05$), while means associated with different letters demonstrate statistically significant differences ($p < 0.05$). Lowercase letters are used to denote statistically significant differences between groups within the same harvest, whereas uppercase letters indicate statistically significant differences between harvests.

Table S2. Toxic metal content of bee venom

Variant		Pb(μg/g)	Cd(μg/g)	Cr(μg/g)
V1	Ss	nd^{dB}	3.061 ± 0.491^{bcA}	2.252 ± 0.639^{aA}
	Rnp1	11.990 ± 2.125^{abA}	2.817 ± 0.138^{aA}	nd^{d}
	Anp1	13.333 ± 3.063^{bA}	nd	3.364 ± 0.727^{cA}
	Snp1	5.236 ± 0.1951^{bB}	nd	2.650 ± 0.411^{cA}
V2	SsB	2.471 ± 0.341^{cC}	2.209 ± 0.217^{cdA}	1.244 ± 0.185^{bC}
	Rnp2	12.660 ± 1.914^{abB}	1.249 ± 0.107^{bB}	nd^{d}
	Anp2	23.144 ± 5.019^{abA}	nd	4.011 ± 0.242^{cA}
	Snp2	4.239 ± 1.93^{bC}	nd	2.182 ± 0.104^{cB}
V3	SsT	nd^{dB}	3.971 ± 0.422^{aA}	2.706 ± 0.314^{cC}
	Rnp3	nd^{cB}	3.067 ± 0.537^{aB}	2.631 ± 0.120^{bB}
	Anp3	36.176 ± 5.662^{abA}	nd	3.255 ± 0.387^{cB}
	Snp3	5.062 ± 0.618^{bB}	nd	4.514 ± 0.408^{abA}
V4	SsO	nd^{dC}	1.971 ± 0.422^{deA}	nd^{c}
	Rnp4	9.964 ± 0.988^{aB}	1.288 ± 0.362^{bA}	1.573 ± 0.180^{cB}
	Anp4	25.876 ± 4.223^{abA}	nd	4.155 ± 0.524^{bcA}
	Snp4	9.988 ± 1.179^{aB}	nd	4.602 ± 0.234^{aA}
V5	SsL	nd^{dB}	1.304 ± 0.316^{ef}	nd^{c}
	Rnp5	13.108 ± 2.165^{abA}	nd	2.934 ± 0.447^{abB}
	Anp5	13.679 ± 6.293^{bA}	nd	5.559 ± 0.499^{aA}
	Snp5	1.155 ± 0.164^{cB}	nd	2.682 ± 0.155^{cB}
V6	SsC	nd^{dB}	3.490 ± 0.205^{ab}	nd^{c}
	Rnp6	16.292 ± 2.63^{aA}	nd	3.401 ± 0.297^{aA}
	Anp6	17.738 ± 3.312^{bA}	nd	1.859 ± 0.226^{dB}
	Snp6	1.668 ± 0.263^{cB}	nd	3.727 ± 0.369^{abA}
V7	SsE	4.901 ± 0.279^{aBC}	0.303 ± 0.056^{g}	nd^{c}
	Rnp7	9.922 ± 1.605^{bB}	nd	1.724 ± 0.194^{cC}
	Anp7	26.783 ± 6.448^{abA}	nd	5.026 ± 0.542^{abA}
	Snp7	9.130 ± 1.650^{cC}	nd	3.961 ± 0.695^{abB}
V8	SsPO	3.373 ± 0.412^{bBC}	0.548 ± 0.108^{fg}	nd^{c}
	Rnp8	9.477 ± 3.736^{bB}	nd	1.673 ± 0.160^{cC}
	Anp8	16.553 ± 3.081^{bA}	nd	5.980 ± 0.322^{aA}
	Snp8	nd^{cC}	nd	3.704 ± 0.504^{bB}

Means marked with the same letters show no statistically significant differences ($p > 0.05$), while means associated with different letters demonstrate statistically significant differences ($p < 0.05$). Lowercase letters are used to denote statistically significant differences between groups within the same harvest, whereas uppercase letters indicate statistically significant differences between harvests.

Nd – Not detectable
